# Hospital Attendants and Male Servants

**Published:** 1905-01-14

**Authors:** 


### HOSPITAL ATTENDANTS AND MALE SERVANTS.

A Hospital Secretary writes: Sir,—On behalf of the Managing Committee, I would beg you to find room in your paper for the insertion of the following, which bears upon a subject of great interest to all hospitals.

During the month of December an Inland Revenue Form, No. 132, "Declaration for Local Taxation (Establishment, etc.) Licences," was forwarded here to be filled up for the year 1904, the suggestion made by the local Revenue officer being that one or more of the men engaged in the necessary service of this institution come within the "definitions" for *male servants* given on said form.

The contention of my committee is that all the men engaged here are employed on duties connected with the work and daily routine of the hospital, and of the patients therein, and not in any exclusive capacity, and, acting under my committee's instructions, I have made a return to that effect.

We should be glad to hear through your valuable columns whether similar claims are made in the case of other hospitals, either in London or in the provinces, and, if so, what is the nature of the return made from those institutions.

